# *E. coli* Meningitis Presenting in a Patient with Disseminated *Strongyloides stercoralis*

**DOI:** 10.1155/2013/424362

**Published:** 2013-11-13

**Authors:** Juliana B. Gomez, Yvan Maque, Manuel A. Moquillaza, William E. Anicama

**Affiliations:** ^1^Department of Internal Medicine, Guillermo Almenara Irigoyen National Hospital, Lima, Peru; ^2^Grupo de Investigacion en Inmunologia, Universidad Nacional de San Agustin, Arequipa, Peru; ^3^Department of Neurology, Guillermo Almenara Irigoyen National Hospital, Lima, Peru; ^4^Department of Pathology, Guillermo Almenara Irigoyen National Hospital, Lima, Peru

## Abstract

*Introduction*. Spontaneous *Escherichia coli* meningitis is an infrequent condition in adults and is associated with some predisposing factors, including severe *Strongyloides stercoralis* (SS) infections. *Case Presentation*. A 43-year-old Hispanic man, with history of travelling to the jungle regions of Peru and Brazil two decades ago, and who received prednisone due to Bell's palsy for three weeks before admission, presented to the Emergency Department with diarrhea, fever, and hematochezia. A week after admission he developed drowsiness, meningeal signs, abdominal distension, and constipation. A cerebrospinal fluid culture showed extended spectrum **β**-lactamase producing *E. coli*. A colonoscopy was performed and showed pancolitis. Three days after the procedure the patient became unstable and developed peritoneal signs. He underwent a laparotomy, which ended up in a total colectomy and partial proctectomy due to toxic megacolon. Three days later the patient died in the intensive care unit due to septic shock. Autopsy was performed and microscopic examination revealed the presence of multiple *Strongyloides* larvae throughout the body. *Conclusion*. *Strongyloides stercoralis* infection should be excluded in adults with spontaneous *E. coli* meningitis, especially, if gastrointestinal symptoms and history of travelling to an endemic area are present. Even with a proper diagnosis and management, disseminated strongyloidiasis has a poor prognosis.

## 1. Introduction

Spontaneous *Escherichia coli* meningitis constitutes about 1-2% of all meningitis cases [1, 2]. Most patients have some predisposing factors, including previous infection by *Strongyloides stercoralis* (SS) [3]. There are different types of presentation of SS infection, ranging from asymptomatic disease until potentially fatal forms like the hyperinfection syndrome with dissemination [2]. Here we report a case of an adult with *E. coli *meningitis associated with disseminated strongyloidiasis. 

## 2. Case Presentation

**Figure 1:**
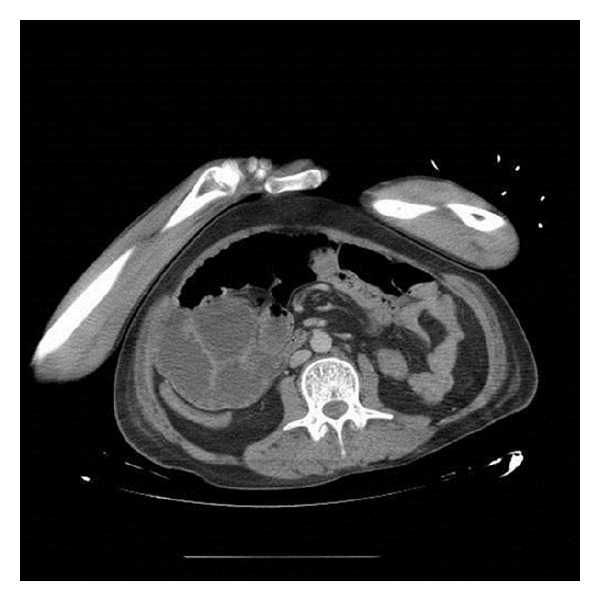
Dilatation of the ascending colon and diffuse thickening of the walls of the transverse colon, which shows partial stenosis and inflammatory changes in the adjacent mesocolon.

The patient was a 43-year-old Hispanic man from Peru, with a history of trips to the jungle regions of Peru and Brazil two decades ago. He was diagnosed by his primary care physician with Bell's Palsy three weeks before being admitted and was started on prednisone 50 mg bid, which he continued to take up to three weeks without proper follow-up. He presented to the Emergency Department with a history of diarrhea, weight loss of 10 kg, and intermittent fever for the three previous weeks. Additionally, the patient presented hematochezia three days prior to admission. He had pallor, tachycardia, mild abdominal pain upon palpation, and blood on rectal examination. The rest of the physical exam was otherwise unremarkable at the Emergency Department. His complete blood count showed 16000 leukocytes/mm^3^ (82% neutrophils, 2% eosinophils, 7% bands, 8% lymphocytes, and 1% monocytes), hemoglobin 8 g/dL, and 700 000 platelets. The patient was suspected to have diverticulitis. He was started on intravenous Ciprofloxacin and Metronidazole, received blood transfusions, and was transferred to an Internal Medicine Service. One week after admission, the patient presented with drowsiness, bradylalia, bradypsychia, nuchal rigidity, positive Kernig and Brudzinski signs, and fever. Furthermore, he developed crackles at the right lung base, abdominal distension, and constipation. A lumbar puncture showed a cloudy and turbid fluid, protein levels 179 mg/dL, glucose 5 mg/dL, cell count 2070 leukocytes/mm^3^ (92% neutrophils, 2% mononuclear cells), and Gram-negative rods in the Gram stain. A new complete blood count showed 25000 leukocytes/mm^3^ (84% neutrophils, 0% eosinophils, 7% bands, 4% lymphocytes, and 5% monocytes). A CT scan of the abdomen revealed a dilated ascending colon (Figure 1).

**Figure 2:**
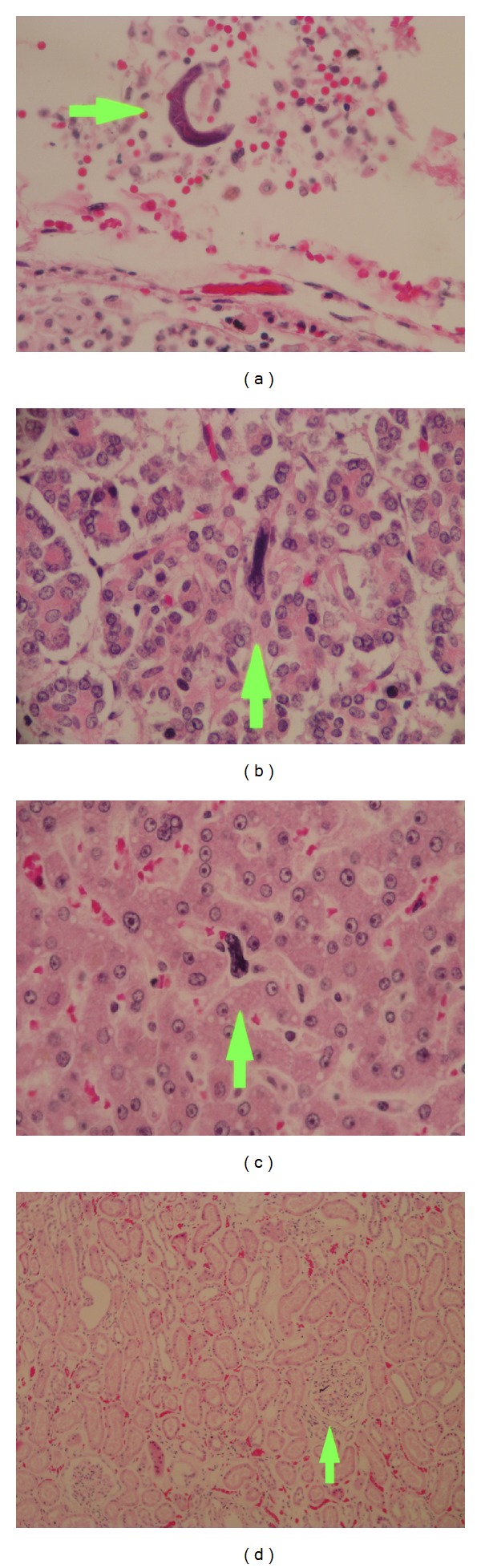
(a) Filariform larvae of *S. stercoralis* longitudinally sectioned in lung tissue, (b) in thyroid tissue, and (c) in liver tissue; the arrows point the larvae, hematoxylin and eosin staining, magnification ×40. (d) Glomerulus with a filariform larva of *S. stercoralis *longitudinally sectioned, hematoxylin and eosin staining, magnification ×10.

He was started on Ceftriaxone and Vancomycin as empiric therapy for meningitis. The CSF culture was positive for extended spectrum *β*-lactamase producing *Escherichia coli*; thus the treatment was changed to Meropenem and Amikacin according to the antibiogram results. After 5 days of treatment, the patient showed improvement of his mental status, the fever remitted, and the abdominal distension decreased; however, the constipation persisted, and this impeded the collection of a stool sample for culture at that point. Initial blood cultures were negatives. The leukocytes values normalized. ELISA test for HIV was performed with a negative result. HTLV test was not performed due to logistic problems in the Hospital Laboratory. Once the patient was stable and the constipation was resolved, he underwent colonoscopy, as the etiology of the hematochezia remained unknown. Results indicated pancolitis and suggested to rule out ulcerative colitis, parasitosis, and tuberculosis. Three days after the procedure the patient developed severe abdominal pain and distension associated with rigidity and guarding. He also presented fever and leucocytosis. Due to suspicion of colonic perforation, he underwent emergency laparotomy. A total colectomy and partial proctectomy were performed due to toxic megacolon, and 300 cc of purulent fluid was found in the abdominal cavity. After the procedure, the patient was admitted to the ICU. New blood cultures were obtained and were positive for extended spectrum *β*-lactamase producing *Escherichia coli*. The patient died three days after the surgery due to septic shock. Autopsy was performed, and microscopic examination revealed the presence of multiple *Strongyloides* larvae in the lungs, trachea, stomach, small and large intestines, thyroid, kidneys, and liver, as well as moderate chronic inflammation of the brain parenchyma and the meninges (Figure 2).

## 3. Discussion

Spontaneous *E. coli *meningitis, which excludes cases related to head trauma, neurosurgical procedures, lumbar puncture, or cerebrospinal fluid cranial fistula, is an infrequent infection in adults (1-2%) [1, 2]. It is usually associated with a poor prognosis, reaching approximately 90% of mortality [4]. 

Common predisposing factors for Gram-negative bacillary meningitis include chronic alcoholism, liver cirrhosis, neoplasia, diabetes mellitus, corticosteroids or other immunosuppressive drugs, HIV infection, and, rarely, systemic infestations with SS [1, 5]. The extended spectrum *β*-lactamase producing *Escherichia coli *causing our patient's meningitis was most likely hospital-acquired since he developed the infection after being hospitalized for several days.

In the present case, the fact that the patient's gastrointestinal disturbances concur with the usage of systemic corticosteroids was underestimated. Since he did not have any other known risk factors, the unusual meningitis was attributed to the corticosteroids use, and the possibility of being related to a parasite infection was not considered.

*Strongyloides stercoralis *is a nematode transmitted from the soil and affects 30–100 million people in the world, mainly in tropical and subtropical areas like Peru and Brazil [6, 7]. The clinical spectrum of this parasite encompasses up to five different presentations, including acute infection with Loeffler's syndrome, chronic infection, asymptomatic autoinfection, symptomatic autoinfection, and hyperinfection syndrome (HS) with dissemination [7].

Most cases of SS infection are asymptomatic or present with mild nonspecific gastrointestinal disturbances. However, hyperinfection syndrome, which occurs due to disruption of the intestinal wall by the parasite and its dissemination through the body, can actually be life-threatening [7, 8]. Identifying SS is challenging since it usually has an irregular larval output. In addition, stool examinations have a low parasite load and immunodiagnostic assays may be ineffective in detecting disseminated infections [6, 9].

It is common among patients who develop hyperinfection syndrome the recent usage of corticosteroids and other immunosuppressive drugs, as well as the presence of other comorbidities like HTLV-1 or HIV infection, alcoholism, and diabetes [6, 7].

Unlike other nematodes, SS is capable of reinfecting the host without the need of an external cycle. This mechanism, known as autoinfection, allows the parasite to persist even for decades in the host [10]. It is highly probable that our patient acquired the parasite when he went to the jungle and remained asymptomatic for many years. Corticosteroid therapy in patients with asymptomatic SS infection can lead to severe forms of the disease as it did in our patient [6]. 

The autoinfection cycle of SS allows it to cause both parasitic and enteric bacterial infections at distant locations in the body, causing localized infections and sepsis by translocation on the nematode surface [3, 11]. In the presented case, SS larvae were found in different organs but not in the brain, where chronic inflammation was the main finding.

Eosinophilia is usually not seen in HS, as in our patient, but in his situation the use of corticosteroids probably also contributed to this fact [12]. 

Different studies report that patients with symptomatic strongyloidiasis have a high frequency of invasive infections by enteric bacteria and *Candida* species, causing sepsis, meningitis, pneumonia, and peritonitis. Most cases occurred in the setting of hyperinfection syndrome with dissemination [3, 13]. In our patient scenario, the parasite presence was not recognized on time, and therefore he did not receive the appropriate treatment and developed *E. coli* meningitis and sepsis, which led to his death.

## 4. Conclusions

Due to the patient epidemiological history and gastrointestinal manifestations, such as weight loss, diarrhea, hematochezia, and ileum, SS infection should have been considered in the differential diagnosis. Clinicians should have a high level of clinical suspicion for the diagnosis of strongyloidiasis in patients with severe extraintestinal infections by enteric organisms without a clear underlying predisposing factor [3, 9]. If a patient is found to have SS and a concomitant serious bacterial infection, an aggressive treatment is warranted. However, even with a proper diagnosis and management, it should be noticed that the prognosis remains poor, since HS can reach a mortality of 76% [14].
